# Sister Death, Cousin Grief: Modes of Presence Before Life and Death

**DOI:** 10.3389/fpsyg.2021.598096

**Published:** 2021-06-03

**Authors:** Roberto Bartholo, Domício Proença Júnior

**Affiliations:** ^1^Laboratory Technologies, Dialogues and Sites, Production Engineering Program, Coppe, Federal University of Rio de Janeiro, Rio de Janeiro, Brazil; ^2^Group for Strategic Studies, Production Engineering Program, Coppe and Public Policies, Strategies and Development Program, IE, Federal University of Rio de Janeiro, Rio de Janeiro, Brazil

**Keywords:** life, death, birth, grief, COVID-19

*Ars longa, vita brevis, occasio praecepts, experimentum periculosum, iudicium difficile*

Art is long, life short, opportunity fleeting, experimentation perilous, and judgment difficult.

– Hippocrates, *Aphorisms*

Like all humans in times of plague or catastrophe, contemporary humanity finds itself before a flood that overflows the dam, demanding answers happy modernity cannot give (Lossky, 1944; Barth, 1946; Glatzer, 1961; Ariès, 1977; Schuon, 2003; Ratzinger, 2020). Individuals are forsaken, to endure death and mourning alone. This much, the Covid-19 Pandemic has exposed.

Happy modernity stands for the contemporary expectations of limitless prosperity and progress, in the shade of unrealized, arguably unrealizable libertarian or socialist promises as well as the recurrent discovery of the unintended and harmful consequences of pursuing boundless progress and prosperity. Every so often, this dream of happy modernity turns into a nightmare, with the irruption of something that exposes its certainty. When a nightmare comes, happy modernity can only offer the promise of ever-more prosperous and progressive answers – and, as these fail, fall silent.

Within it lies also, more prominent if less pronounced, an illusion: that happy modernity’s prosperity and progress will lead to a humanized nature, to a world where medicalization would tame death and expel it from life, building an invincible dam against suffering. The presumed immortality of institutions could lend this permanence to the contingency of individual life, for the price of accepting anonymous interchangeability and blurring of boundaries between private and public. Individual existence would be abstracted as part of a given, momentary and transient totality, a pseudo-transcendental whole that would contain all meaning, possess all value, express all sense – and endure “forever” or until the next correct narrative, whichever comes first. Once again, reality shows that the pretension of expelling death from life is vain; worse, it is misleading. There is an irredeemable individual cost in accepting the illusion, in believing this offer.

Franz Rosenzweig (1886–1929; a *morenu*, “one of our teachers,” a traditional Jewish rabbinic distinction) wrote *Der Stern der Erlösung*, the *Star of Redemption* (Rosenzweig, 1921). It is one of the oddest of war books. *Unteroffizier* Rosenzweig wrote it while serving the Austro-Hungarian Army on the Balkan Front in World War One. It was written while he was a participant in this *small Jihad* (in the breaks of the marches, of battles, of holding positions, of the hospital, of the retreat). His *magnum opus, Der Stern* is essentially his testimony and commitment to the *great Jihad*, the struggle against the annihilation of the person by philosophical systems that aim to dissolve individual existences.

The circumstantial, momentary, foremost threat at his time was German Idealism, crowned by Hegel’s philosophy and its attempt to unveil the “essence” of the world, to arrive and grasp at certainty. An attempt rooted in a fear of living, a vain effort to elude death. It would offer a refuge in abstraction, in the peaceful dissolution of all selves in a whole, mobilizing a cognition that has an end in itself, being afraid of life and all the disturbances and uncertainties life brings. *Der Stern* is a strong rebuttal of any such attempt. Life is, as sung in *La Traviata* (Verdi, 1853): *croce e delizia al cor* (a cross *and* a delight for the heart), personal for each individual.

*Der Stern* ends with these words: “into life”! a farewell to books and academic concepts and theories. Into life, free from being taken as disturbance on a system, forsaking all systems, because any system hinders the plenitude of life as an inexhaustible fountain of renewal. *Erkennen* (cognition) is no longer an end in itself, it has turned into a service, it has become an ancilla for life, that only gains purpose when called upon. Such a cognition is a friend of living life and attends to it in all its needs, foibles and consequences.

*Gesunder Menshenverstand in Handeln* (sound common sense in action) is how Rosenzweig (1935) calls his method, as presented in *Das Büchlein vom Gesunden und Kranken Menschenverstand* (Rosenzweig, 1925). The *Little Book on Healthy and Sick Human Understanding*, published posthumously (translated as *Understanding the Sick and the Healthy, a view of World, Man, and God*). Rosenzweig offers the foundation for a *Neues Denken* (new thinking) critical of philosophy conceptualized. It is built on the bare acknowledgment that cognition is not independent of time, on the contrary, it is bound at each moment to that moment, cannot undo its past, cannot avoid its future.

Strictly speaking, this “new thinking” is grammatical, not merely logical. It is living, “speaking thinking” that needs someone else to take place, that can call upon cognition for this one living, dialogical, relational purpose. Its root is a radical gamble upon the value of living experience, in thinking of life living – lived, not conceptualized, so that it encloses the widest variety of relational exchanges – with other humans, with the world, with the divine. Rosenzweig’s “new thinking” amounts to a veritable *philo-zoe* (zoe, life), signaling the counterpoint with philo-sophy. Philo-zoe includes philo-sophy as one among other dimensions of relational experience.

Wonder is at the heart of it all: the awe before marvels and estrangements. Estrangement, marveling, wonder have long been recognized as a door to philosophy. The problem begins if this access is presented as exclusive, even proprietary, as if the sole virtuous purpose of wonder was to lead to the path of abstract philosophizing. Most damning of all: to lead to a philosophical removal of wonder from life.

Rosenzweig’s call, “into life”! frees wonder from the chains of conceptual abstractions. It acknowledges a wide variety of possible doors, leading to various paths of marvelings and estrangements, not denying that one among such doors would lead to philosophical paths.

Freeing wonder from philosophical claims of eternity and exclusivity, philo-zoe acknowledges birth and death as the paramount marvels of life. It is love, human love, living love that spans from the marvel of birth to the marvel of death, accepting the newborn from the moment it is to come – in expectation – and retaining the bond with the dear departed – in grief.

To be born is to arrive; it engenders a world anew, posing to the living needs of receiving and of being received. Birth is welcome and commitment to an otherness, since no human newborn survives alone. Newborns entail relational imperatives: of the world with them, of each newborn with the world. The joys of expectation and birth are modes of love, homage to the surprises, demands and potentials of a new presence.

Conversely, to die is to leave. For each dead, one world stops. Only the living remains. And their worlds endure with them. Grief is homage to a memory. To grieve is to make that memory living. The longing of grief is a mode of love, nostalgia for the departed, wishful for a presence made impossible by death – left only in the memory of those still living.

The acknowledgment of the marvels of birth and death are thus lovingly intertwined as *croce e delizia al cor* with the longings of expectation and grief. This is what sound commonsense in action may offer: access to the fruition of each mortal life – exciting, boring, painful, joyful, tearful, hopeful, despairful; particular, individual, unique; and, uppermost, finite. This means: bound in time through experience, from birth to death, the final alterity. But these are the paths the abstractions of “pure” philo-sophy fail to acknowledge, as Rosenzweig tells us.

We shared in this brief essay a mode of thinking that argues for philo-zoe rather than philo-sophy. This would be a mode of thinking for the lover of wisdom *lived*, loving living, rather than of wisdom *abstracted and conceptualized*. Philo-zoe offers, stands for, spouses an acknowledgment of the facticity of human experiences as possible meetings in time. Each meeting has its due time, occasion, span, density, intensity, purpose – duration during the time each of us lives and we can meet. Meetings mean encounters and dialogs with others, with the world, with the divine. Hence, also with “brother birth” and “sister death,” that ensure life’s temporality with the seal of mortality; and in anticipation of potential joy of an incoming alterity, with “cousin expectation,” or in apologetical rite for alterities passed away, with “cousin grief” too.

As poets have told us –

Everytime we say goodbye, I die a little

Everytime we say goodbye, I wonder why a little

Why the Gods above me, who must be in the know

Think so little of me, they allow you to go

*Every time we say goodbye I die a little*, Cole Porter

*Warum ist Wahrheit fern und weit?* | Why is Truth so far and away?

*Birgt sich hinab in tiefste Gründe?* | Hiding in the deepest abyss?

*Niemand versteht zur rechten Zeit!* | No one understands at the right time!

Hikmet Nameh. Buch der Sprüche, in *West-östlicher Diwan*, Goethe

## Author Contributions

Both conceived, drafted, and revised the manuscript together.

## Conflict of Interest

The authors declare that the research was conducted in the absence of any commercial or financial relationships that could be construed as a potential conflict of interest.
